# Study about evaluation of efficacy of methotrexate in localized scleroderma using ultrasonography

**DOI:** 10.1111/srt.13300

**Published:** 2023-03-02

**Authors:** Fan Zhang, Jianke Li, Qing Zhao, Hong Liu, Furen Zhang

**Affiliations:** ^1^ Shandong Provincial Institute of Dermatology and Venereology and Provincial Hospital for Skin Diseases Shandong First Medical University and Shandong Academy of Medical Science Jinan China

**Keywords:** evaluate, methotrexate, morphea, ultrasonography

## Abstract

**Background:**

The treatment and curative effect evaluation of localized scleroderma (LS) still perplexes many clinical workers.

**Purpose:**

To investigate the efficiacy of methotrexate in the treatment of LS by the evaluation of ultrasonography.

**Methods:**

A prospective study enrolled 10 patients treated with MTX for at least 6 months was conducted. Treatment outcome was evaluated by a clinical score and 15‐MHz ultrasonography. Safety assessment included the monitoring of adverse drug reactions and clinical laboratory examinations.

**Results:**

Eight of the 10 patients achieved clinical remission only with MTX. One patient was relieved after MTX combined with corticosteroids, while another one does not improve after the treatment of mycophenolate mofetil and corticosteroids. The effective rate of MTX is 80%. Nine patients were significantly improved with a decrease of the Localized Scleroderma Cutaneous Assessment Tool (the mean score of the LoSCAT cutaneous activity dropped from 5.2 to 1.0, *p* < 0.001, the mean score of the LS cutaneous damage dropped from 4.3 to 2.3, *p* = 0.002). The average difference of thickness between skin lesions and normal skin evaluated by ultrasonography decreased from 0.13 cm to 0.04 cm (*p* = 0.009) in eight patients. No serious adverse reactions occurred.

**Conclusion:**

Methotrexate is a safe and effective treatment for patients with LS. Ultrasonography can be considered as an efficient assessment tool for evaluation LS.

## 1 INTRODUCTION

Morphea, or localized scleroderma (LS) is a group of idiopathic sclerotic skin disease characterized by thickening and sclerosis of skin, including plaque, linear, generalized, deep, pansclerotic scleroderma, and mix scleroderma.^1^ The annual incidence of LS is 3.4–27 cases per 100 000 population, and female plays a dominant role, with a ratio of 2.6: 1.^2^, ^3^, ^4^ Although several cases undergoing spontaneous remission, many patients may develop joint contractures, deformity, and severe functional impairment in children.^5^ Early treatment is crucial to prevent progression of the disease. For most clinicians, the treatment of LS is still a challenge. Immunosuppressive therapy is used to treat LS patients.^6^ Methotrexate (MTX) in the treatment of LS has been widely reported in many countries.^7^ However, in China, traditional Chinese medicine to improve vascular microcirculation is the first choice for most doctors, such asasiaticoside tablets, Danggui Sini decoction and so on these traditional Chinese medicine treatments rely on experience without sufficient experimental evidence. Meanwhile, accurate assessment tools for evaluation of severity and efficacy of scleroderma are not available. Durometer, thermography, magnetic resonancehas, and other physical measurement tools have been used for the evaluation of scleroderma. The efficiency of these tools is uncertain.^8^, ^9^, ^10^, ^11^, ^12^, ^13^ Ultrasonography, as a cheap and convenient detection tool, has been used to evaluate the treatment of a variety of sclerosing skin diseases. In particular, the change of lesion thickness measured by ultrasonography can well reflect the therapeutic effciacy. Thus, we conducted a study to: (1) explore the efficacy of MTX in the treatment of LS in China; (2) evaluate the efficiency of ultrasonography in monitoring the thickness of skin lesions in LS patients.

## 2 MATERIAL AND METHODS

### 2.1 Patients

From May 2020 to October 2021, a single‐center nonrandomized prospective cohort study was conducted on patients with LS in the Shandong Provincial Hospital for Skin Diseases. A total of ten patients were included in the study. The diagnosis of LS was made according to the clinical manifestations and the histopathological examination. All patients met the following inclusion criteria: (1) patients with active skin lesions (active skin lesions are defined as enlarged preexisting skin lesions, new skin lesions, or inflammatory skin lesions) or linear skin lesions in face; (2) the patient was untreated before visiting our hospital. Exclusion criteria included: (1) patients with abnormal blood routine or liver and kidney function test results before treatment; (2) the patients who was complicated by systemic diseases; (3) pregnant women or patients with fertility requirements. Detailed information was collected, including demographic information (sex, age, personal, or family medical history), clinical information (type of LS, the period before diagnosis, laboratory tests’ results before initial treatment, dose of MTX, adverse events during treatment, treatment duration, treatment response, etc.). The skin lesions were evaluated every 3 month and blood routine, liver and kidney function tests were examined to prevent adverse reactions. This study was approved by the ethic committee, and all patients signed informed consent.

### 2.2 Treatment protocol

**FIGURE 1:**
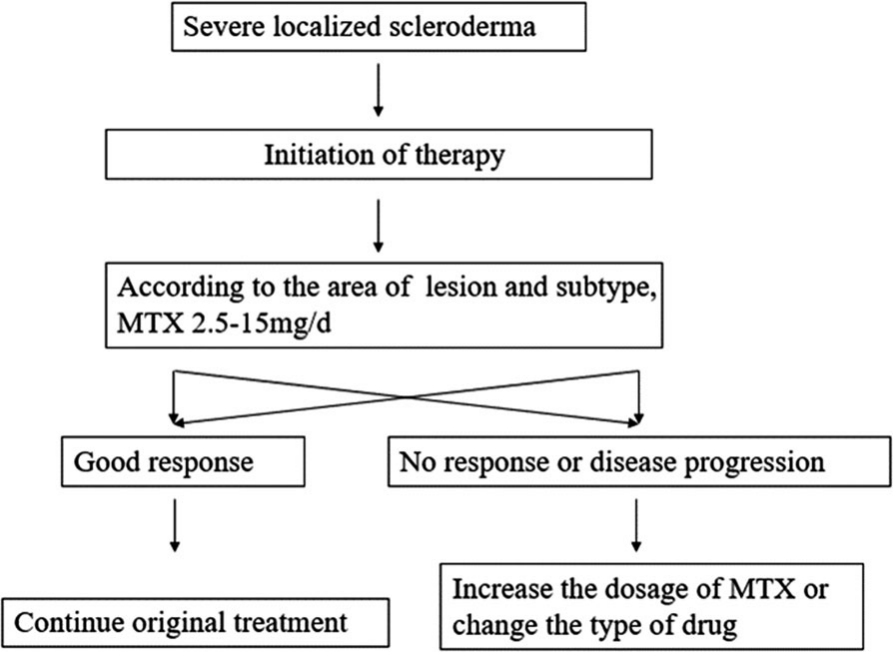
Treatment algorithm for nine patients.

Initially, patients were treated with MTX alone. For patients between 5 and 85 years, 2.5–15 mg/week of MTX were given according to the patient's weight and the extent of the skin lesions. If no improvement was observed for eight consecutive weeks, new skin lesions appeared, or preexisting lesions expanded within 4 weeks, the therapeutic regimen changed as follows: increase the dose of MTX or MTX combined with prednisone 5–10 mg/day or substituted MTX for mycophenolate. The flow chart has been provided in Figure 1. If the patient has adverse reactions in the course of treatment, the drug dose may be adjusted.

### 2.3 Clinical outcomes evaluation

#### 2.3.1 The LS cutaneous assessment tool (LoSCAT)

Patients are evaluated by the same dermatologist at every follow‐up visit. LoSCAT,^12^ which consists of an activity index LoSAI and a damage index LoSDI as well as ultrasonography, was used as measures’ tools. Additionally, we have collected clinical photos of patients at every visit, to facilitate the assessment of the improvement in the skin lesions.

#### 2.3.2 Ultrasonographic evaluation

Ultrasonography was performed on the representative lesions of every patient to assess dermal thickness with a digital 15‐MHz ultrasonography scanner by two radiologists. For generalized lesions, we select one of the most serious lesions as the representative to measure, and a single lesion does not need to be selected. The penetration depth of this instrument is greater than or equal to 30 mm. At the same time, the normal skin on the contralateral side or around the lesion was also measured as control. In order to reduce the measurement error and control variables as much as possible, we controlled the air conditioning temperature in the ultrasonic room so that the ambient temperature was kept between 20 and 23°C. And, the patient rests for at least 5 min before the examination. The examiner pressed the patient's skin vertically and evaluated the thickness, echo intensity and blood flow signal of the patient's epidermis, dermis, and subcutaneous tissue, comparing it with the surrounding or contralateral normal skin. Then the difference between normal skin and affected skin was calculated. According to the ultrasonography results before and after treatment, we defined the results as follows: (1) complete response (CR) indicated the improvement rate of ultrasonography results reached more than 95%, and the skin lesions almost completely disappeared; (2) partial response (PR) was defined as the hardness of the skin lesion has improved, and the improvement rate of the ultrasonography score was >0% and <95%; (3) no response: the area, hardness, and atrophy of involvement had no change; (4) worsening: the difference value of thickness between lesion and normal skin has increased.^9^ For some insurmountable reasons, the frequency of ultrasonographic examination was not measured once a month in 10 patients, but each patient was measured at least twice during the experiment.

### 2.4 Statistical analysis

Data of clinical scores and difference of thickness between lesions and normal skin evaluated by ultrasonography were given as the standard deviation of mean plus minus. Normal distribution of collected data was analyzed by the Shapiro‐Wilk test, while the association between LoSCAT and skin thickness measured by ultrasonography was tested by the Spearman correlation coefficient. Measurement data before and after treatment were examined by paired‐samples T test. *p* < 0.05 was considered to be significant. Statistical analysis was performed by SPSS.

## 3 RESULTS

### 3.1 Patient

Ten patients were included in this cohort, and the characteristics of patients are showed in Table 1 and Table S1. The average age of the ten patients was 39.3 ± 24.6 years. Among them, four patients (40%) were linear face/scalp, four patients (40%) were generalized, and two patients (20%) were plaque type. The location of skin lesions included head, face, trunk, and limbs. Skin atrophy and alopecia were seen in patients with linear scleroma, while a case of generalized morphea patients suffered from limited joint movement. The average diagnostic delay was 3.3 ± 4.7 year. Two patients had positive antinuclear antibody test. No systemic disease was found in all patients.

**TABLE 1:** Characteristics of patients.

Patient number/sex/age	Subtype	Diagnostic delay (year)	Affected areas	Associated morbidity	Previous treatment	Diagnosis method	ANA
1/F/20	Linear face/scalp	5	Forehead	Alopecia;skin sag	None	Biopsy	NA
2/F/11	Generalized	4	Waist; both legs	Skin atrophy; limited joint movement	None	Biopsy	Pos
3/F/53	Linear face/scalp	15	Forehead	Skin sag	None	Biopsy	NA
4/F/18	Linear face/scalp	1	Forehead	Alopecia; skin sag	None	Reflective confocal microscope	Neg
5/F/44	Generalized	0.25	Waist; left leg	Skin atrophy	Topical steroid ointment	Biopsy	Pos
6/F/56	Plaque	1	Abdomen	Skin sag	Topical steroid ointment	Biopsy	Pos
7/F/53	Plaque	0.2	Abdomen	Skin atrophy	None	Biopsy	NA
8/F/51	Linear face/scalp	7	Forehead	Alopecia; skin sag	None	Biopsy	Neg
9/M/82	Generalized	0.5	Abdomen; back; both legs; neck	Pain; skin atrophy	None	Biopsy	Neg
10/F/5	Generalized	0.5	Right upper limb; right axilla; left leg	skin atrophy; joint activity limits	None	Biopsy	NA

Abbreviations: NA: not application

### 3.2 Treatment

Ten patients were treated according to the treatment protocol as shown in Figure 1. All patients received MTX in the study.

### 3.3 Clinical outcomes

#### 3.3.1 LoSCAT

The LoSCAT scores of the nine patients were decreased except patient number 9, whose condition was not observed to improved. The average LOSAI scores of the nine patients before treatment was 5.2, which dropped to 1.0 after treatment (*p* < 0.01). The maximum improvement rate was 100%, and the minimum improvement rate was 40%. The average LOSDI score decreased from 4.3 to 2.3 (*p* = 0.002) after treatment overall.

#### 3.3.2 Ultrasonographic evaluation

**FIGURE 2:**
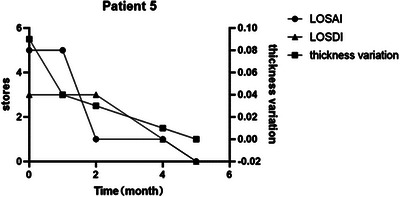
Changes of LoSCAT score and lesion thickness in patient 5.

According to the definition of reaction degree, except for patient number 9, all the other patients had clinical improvement, including seven patients with PR and two patients with CR (Table 2). And the seven patients’ improvement rate of the ultrasonographic evalution was all ≥50%. The difference of dermis thickness between skin lesions and normal skin decreased from 0.13 to 0.04 cm (*p* = 0.009). The average improvement rate of dermis thickness was 69%, the maximum improvement rate was 100%, and the minimum improvement rate was 30%. According to the results of ultrasonography, average first reaction time of nine patients was 1.8 months, which shorter than the earliest response time reported in other study. Being investigated through Spearman correlation coefficient analysis, there was no obvious correlation between the clinical activity score and the skin thickness changes measured by ultrasonography. However, we found that the values measured by ultrasonography changed earlier than the clinical score. It was indicating that ultrasonography was more sensitive for evaluation of dermis thickness. Ultrasonography can often measure thickness changes in skin lesions in a short period of time, which was more pronounced in patients with a shorter history of the disease (Figure 2). Under ultrasonographic monitoring, we did not find the changes of hypervascularity and alterations of the echogenicity of the subcutaneous tissue during the treatment. However, compared with the surrounding normal skin, the echogenicity of the diseased skin decreased or increased.

**TABLE 2:** Detailed treatment and adverse reactions of 10 patients.

			LoSAI		LoSDI		Thickness of skin lesions				
Patient number/Age	Dose of MTX	Duration of treatment (month)	Before	After	Before	After	Before	After	Outcome	Initial response time (month)	Adverse event
1/20	10 mg/w	6	4	1	2	1	0.100	0.070	PR	4	Mild gastrointestinal reaction
2/11	7.5 mg/w ^a^	6	6	0	3	1	0.040	0.010	PR	2	Mild gastrointestinal reaction
3/53	15 mg/w	9	4	1	5	3	0.290	0.080	PR	1	No
4/18	12.5 mg/w	8	4	0	2	0	0.050	0.000	CR	2	No
5/44	10 mg/w	6	5	0	3	0	0.090	0.000	CR	1	A slightly elevation in transaminases
6/56	12.5 mg/w	6	6	1	12	7	0.165	0.105	PR	2	Mild gastrointestinal reaction
7/53	15 mg/w	9	8	2	4	3	0.080	0.040	PR	1	No
8/51	10 mg/w	7	5	3	3	2	0.310	0.060	PR	3	Mild gastrointestinal reaction
9/82	10 mg/w^b^	6	7	7	12	12	–	–	worsening	–	No
10/5	5 mg/w^c^	6	5	1	5	4	0.075	0.015	PR	1	No

^a^ Due to gastrointestinal reactions, the patient adjusted the dose to 7.5 mg after oral 10 mg of MTX for 2 months.

^b^ Because of the bad clinical response, MTX was replaced by mycophenolate mofetil after 2 months.

^c^ After 3 months of treatment of MTX alone, prednisone of 5 mg was increased daily due to adverse clinical reactions.

### 3.4 Adverse events

Four patients had mild gastrointestinal reactions. Three patients were gradually tolerated, and another patient needed to reduce the dose of MTX (from 10 mg/week to7.5 mg/week after 2 months). One patient had a slight increase in transient transaminase, and was treated with compound glycyrrhizin tablets.

## 4 DISCUSSION

**FIGURE 3:**
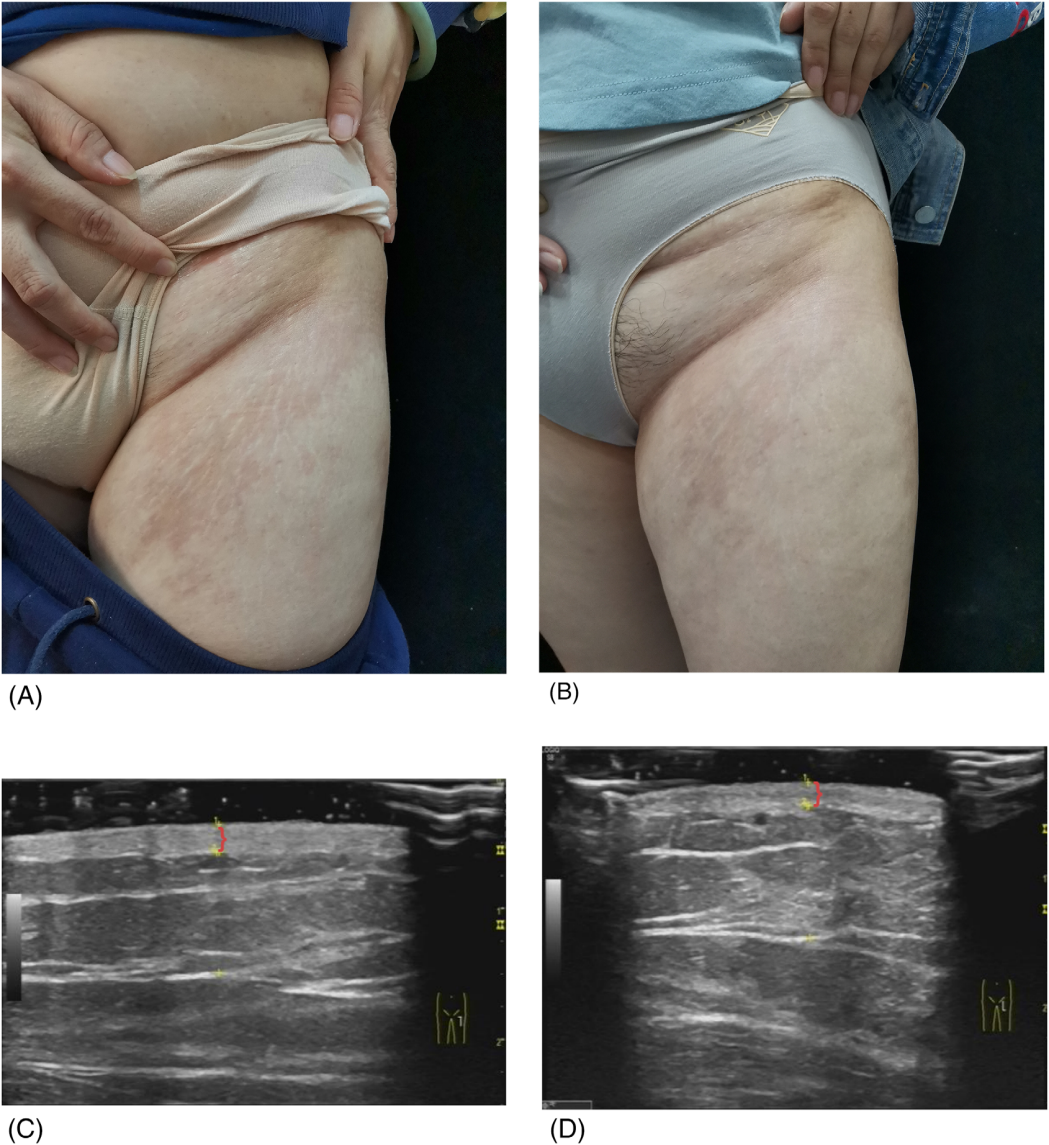
(A and B) Localized morphea lesions on the groin. The hardness, pigmentation, and atrophy of the skin lesions were significantly improved during the 6‐month treatment period. (C and D) Changes of skin thickness (the part indicated by the red bracket) before and after treatment under ultrasonography monitoring (skin thickness decreased from 0.21 cm to 0.18 cm).

**FIGURE 4:**
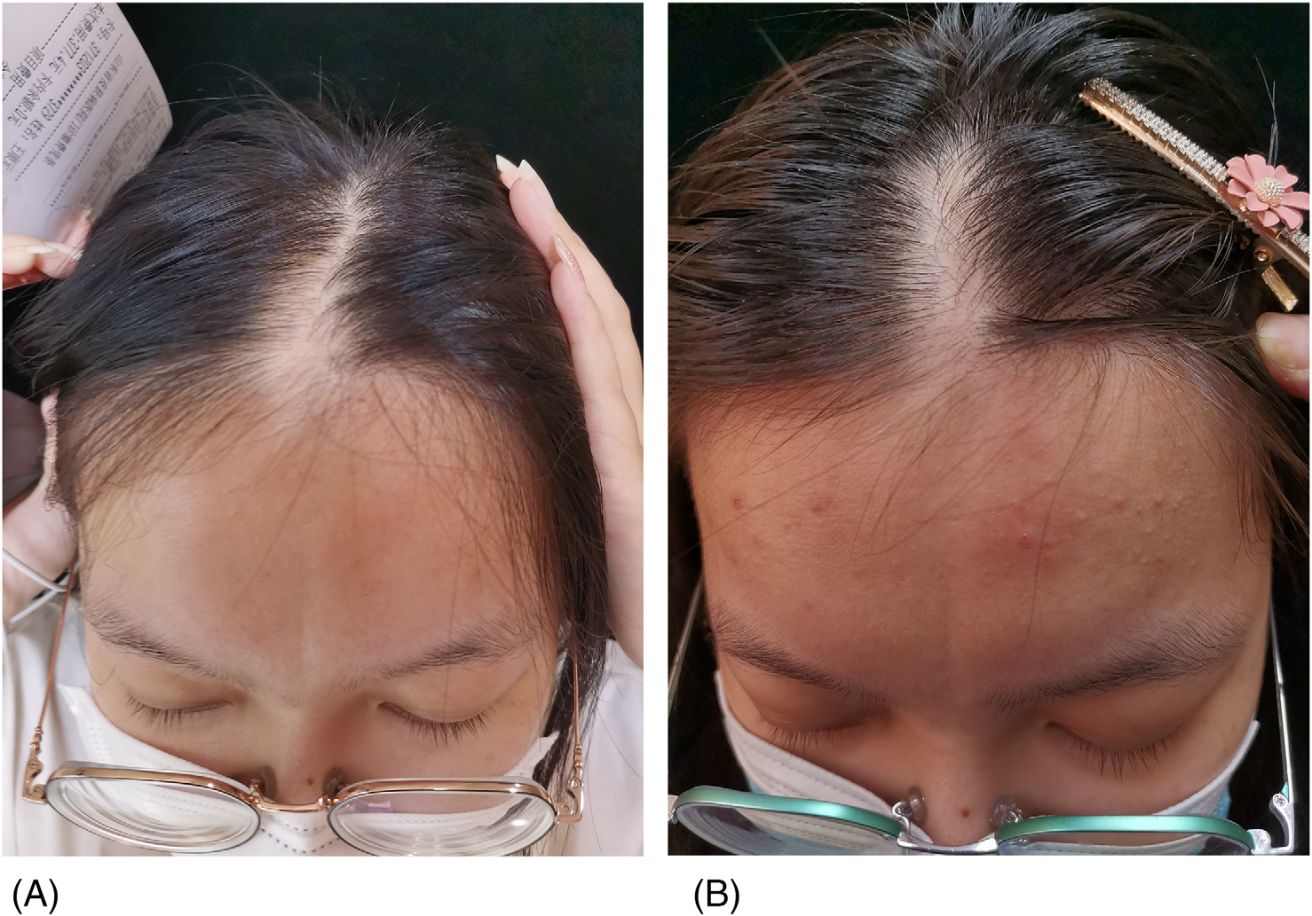
(A and B) En coup de sabre (linear morphea subtype) affecting the forehead and scalp. During the treatment period of 8 months, the hair regenerated and the area of hypopigmented spots decreased.

This study was conducted to evaluate the efficacy of MTX in the treatment of LS by using clinical score and ultrasonography. A total of 10 patients were recruited. Clinical and ultrasonographic evaluation revealed that nine patients markedly improved during 6‐month follow up, and even linear morphea patients were found to have hair regrowth (Figures 3 and 4), showing high efficacy.

As early as 1998, Seyger et al.^12^ evaluated the efficacy of MTX alone in the treatment of LS in adults. Six patients completed the study, and clinical improvement was sufficient in all six patients with 100% efficiency. Other treatments such as MTX combined with corticosteroids have also been proven effective in a randomized controlled study.^14^ For some stubborn lesions, mycophenolate mofetil and hydroxychloroquine have also been tried and verified by a few cohort studies.^11^, ^15^ A prospective comparative effectiveness study of three different methotrexate‐based regimens for juvenile LS found there was no significant difference in efficacy between MTX alone group and MTX combined with corticosteroids treatment group. The total effective rate was 75%.^16^ The researchers also found that patients with treatment failures had a wider range of lesions and higher clinical scores. In our study, eight Chinese patients treated by MTX alone had sufficient clinical improvement, including six patients with PR, and two patients with CR. One patient achieved clinical improvement after MTX combined with corticosteroids. Only one patient had poor clinical response. The effective rate of MTX alone in the treatment of LS was 80%. Two patients with failure to use MTX alone had higher LoSCAT scores at baseline and more extensive skin disease. Overall, the data suggest that MTX may be beneficial for LS.

Up to now, many different tools have been used to evaluate the efficacy of treatment for LS.^17^, ^18^, ^19^ Seyger et al.^12^ used a hardness tester and the unmodified LoSCAT for therapeutic evaluation. The semi‐quantitative score of the autonomous six‐point system was applied to monitor the efficacy.^18^ Initial reaction time and activity were used for assessing the efficacy of MTX in the treatment of children with LS.^8^ However, due to the complexity and subjectivity of these tools, they were not widely used.

Ultrasonography has also been used to assess the therapeutic efficacy in LS.^20^, ^21^, ^22^ Skin thickening can represent either the earlier edema stage or the later fibrosis stage. The thinning of the skin thickness represents a period of atrophy. Therefore, the change of thickness of LS lesions can be used to detect the efficacy of drugs. In our study, we found that there were significant differences between the thickness of the skin lesions before and after treatment. M. Arisi^23^ found no statistical significance in skin thickness evaluated by ultrasonography in LS patients who received ultraviolet A1 phototherapy. This might be due to the 50 MHz parameter he used, which had less penetrating power. In addition, M. Arisi considered that phototherapy might only change the order of collagen fibers without reducing its total number. In our study, the average improvement rate of skin thickness measured by ultrasonography in eight patients after 6 months of treatment was 69%, which was lower than the improvement rate of LoSAI. However, the dermis thickness assessed by ultrasonography has been changed earlier than the clinical scores, indicating that ultrasonographic assessment was more sensitive than the scores, which may aid the doctor to grasp the situation more quickly and take the appropriate treatment program. There was also no correlation between the dermis thickness measured by 14‐MHz ultrasonography and LoSCAT.^24^ Although the changes in the echogenicity and hyperaemia of the skin lesions were hard to obtain and compared in our study, after summing up previous studies, Suzanne C. Li^20^ found that the echogenicity of the skin lesions in the early stage had different degrees of reduction, and blood flow signals were strong, which indicates dermal edema. At the same time, echo enhancement represents the hardening of dermal collagen fibers. LoSCAT was dependent on clinicians whereas ultrasonographic assessment was more objective. Thus, combination clinical score and ultrasonography would be more comprehensive.

The mechanism of MTX in treating LS remains unclear. It is generally believed that the inflammatory pathway included a variety of cytokines and chemokines are involved in the pathogenesis of LS, which is mainly caused by the Th1 cells in the early stage of the disease and the Th2 cell subsets in the late stage of LS, resulting in fibrosis.^25^ Methotrexate plays an anti‐inflammatory role by increasing the concentration of adenosine extra and intracellular, and it is assumed that by adenosine inhibits the oxidative burst of neutrophils and mononucleocytes, prevents the chemotaxis of leukocytes, and inhibits the secretion of multiple cytokines by monocytes and macrophages. Finally MTX interferes with inflammatory pathways in LS.^26^

There are some limitations in our study. Firstly, only 10 patients were involved in our study. Secondly, according to the limited number of patients, various age groups were not separate for discussion. In addition, the follow‐up was only conducted for 6 months. It was suggested that the time of maintenance therapy should be continued for at least 2 years. After stopping treatment, these patients were advised to monitor the condition for at least 5 years.^27^

## 5 CONCLUSION

In conclusion, most patients included in our study showed excellent response to MTX without any major adverse effects. Ultrasonography could be used for monitoring the improvement of dermis thickness. Further studies are needed to evaluate the optimal effective dosage in different subset LS patients.

## CONFLICT OF INTEREST STATEMENT

The author reports no conflict of interest in this work.

## FUNDING INFORMATION

The Academic Promotion Programme of Shandong First Medical University, Grant Number: 2019LJ002,2019RC007; The Youth Technology Innovation Support Project of Shandong Colleges and Universities, Grant Number: 2019KJL003; The Innovation Project of Shandong Academy of Medical Sciences

## Supporting information

Supporting‐Information

## Data Availability

Research data are not shared.
