# Supplementary material for: Study about evaluation of efficacy of methotrexate in localized scleroderma using ultrasonography
Source: Skin Res Technol. 2023 Mar 2;29(3):e13300. doi: 10.1111/srt.13300 (PMC10155795; doi:10.1111/srt.13300)
Supplement: Supplementary file 1 — Supporting‐Information [file SRT-29-e13300-s001.docx]

**Table S1** Clinical information for patients

| **Characteristic** | **Result** |
| --- | --- |
| Age, years, median | 39.3±24.6 |
| Male, n (%) | 1(10%) |
| Disease duration before therapy started, years, median | 3.3±4.7 |
| Type of localized scleroderma |  |
| Linear face/scalp | 4(40%) |
| Generalized | 4(40%) |
| Plaque | 2(20%) |
| Location of skin lesions |  |
| Head | 4(40%) |
| Trunk | 3(30%) |
| Upper limbs | 3(30%) |
| Lower limbs | 4(40%) |
| Systemic autoimmune disease | 0 |
| Positive results of antinuclear antibodies | 2(20%) |
| Increased erythrocyte sedimentation rate | 2(20%) |
